# Reduced hypertrophy in vitro after chondrogenic differentiation of adult human mesenchymal stem cells following adenoviral *SOX9* gene delivery

**DOI:** 10.1186/s12891-020-3137-4

**Published:** 2020-02-17

**Authors:** M. Weissenberger, M. H. Weissenberger, F. Gilbert, J. Groll, C. H. Evans, A. F. Steinert

**Affiliations:** 1 Department of Orthopaedic Surgery, König-Ludwig-Haus, Center for Musculoskeletal Research, Julius-Maximilians-University, Brettreichstrasse 11, D-97074 Würzburg, Germany; 2 Department of Pathology, Caritas-Hospital, Bad Mergentheim, Germany; 3 Department of Orthopaedic Trauma, Hand, Plastic and Reconstructive Surgery, University Hospital Würzburg, Würzburg, Germany; 4 Department of Functional Materials in Medicine and Dentistry, Julius-Maximilians-University, Würzburg, Germany; 5 Rehabilitation Medicine Research Center, Mayo Clinic, Rochester, MN USA; 6 Present address: Department of Orthopaedic, Trauma, Shoulder and Arthroplasty Surgery, Rhön-Klinikum Campus Bad Neustadt, Bad Neustadt a.d. Saale, Germany

**Keywords:** Mesenchymal stem cell, Cartilage, SOX9, Gene therapy, Chondrogenesis, Hypertrophy, Adenovirus, Bone marrow

## Abstract

**Background:**

Mesenchymal stem cell (MSC) based-treatments of cartilage injury are promising but impaired by high levels of hypertrophy after chondrogenic induction with several bone morphogenetic protein superfamily members (BMPs). As an alternative, this study investigates the chondrogenic induction of MSCs via adenoviral gene-delivery of the transcription factor SOX9 alone or in combination with other inducers, and comparatively explores the levels of hypertrophy and end stage differentiation in a pellet culture system in vitro.

**Methods:**

First generation adenoviral vectors encoding *SOX9*, *TGFB1* or *IGF1* were used alone or in combination to transduce human bone marrow-derived MSCs at 5 × 10^2^ infectious particles/cell. Thereafter cells were placed in aggregates and maintained for three weeks in chondrogenic medium. Transgene expression was determined at the protein level (ELISA/Western blot), and aggregates were analysed histologically, immunohistochemically, biochemically and by RT-PCR for chondrogenesis and hypertrophy.

**Results:**

*SOX9* cDNA was superior to that encoding *TGFB1*, the typical gold standard, as an inducer of chondrogenesis in primary MSCs as evidenced by improved lacuna formation, proteoglycan and collagen type II staining, increased levels of GAG synthesis, and expression of mRNAs associated with chondrogenesis. Moreover, *SOX9* modified aggregates showed a markedly lower tendency to progress towards hypertrophy, as judged by expression of the hypertrophy markers alkaline phosphatase, and collagen type X at the mRNA and protein levels.

**Conclusion:**

Adenoviral *SOX9* gene transfer induces chondrogenic differentiation of human primary MSCs in pellet culture more effectively than *TGFB1* gene transfer with lower levels of chondrocyte hypertrophy after 3 weeks of in vitro culture. Such technology might enable the formation of more stable hyaline cartilage repair tissues in vivo.

## Background

Once articular cartilage is injured it has very limited capacity to heal. Mesenchymal stem cells (MSCs) derived from human bone marrow and other sources provide an attractive alternative to chondrocytes for cartilage repair [1]. However, the most appropriate factor or combination of factors to drive MSCs toward chondrogenesis and stable hyaline neocartilage formation remains to be elucidated [2]. We and others have extensively tested several members of the transforming growth factor (TGF)-β superfamily including TGF-β1 (encoded by *TGFB1*), bone morphogenetic protein (BMP)-2, BMP-4, BMP-6 and BMP-7, the fibroblast growth factor (FGF) family or the family of hedgehog proteins (e.g. sonic or indian hedgehog (SHH, IHH)) for their potential to induce chondrogenic differentiation of MSCs in vitro [3–5]. For efficient delivery of such growth factors, we have successfully explored the potential of adenoviral gene delivery of *TGFB1*, *BMP2* or *BMP4*, or *IHH* for efficient chondrogenic induction of MSC aggregate cultures. Although successful in promoting chondrogenesis, these factors also produced high levels of chondrocyte hypertrophy and apoptosis, which was most abundant for *BMP2*, but also present in the *BMP4*, *IHH* and *TGFB1* gene modified cultures [6–8]. Although, insulin like growth factor (IGF) 1 is a known mediator of growth plate development and increases extracellular matrix (ECM) synthesis in chondrocytes, *IGF1* gene delivery could not induce chondrogenesis alone in primary MSCs or enhance chondrogenesis in combination with *TGFB1* or *BMP2* [6]. Chondrogenic hypertrophy of adult MSCs represents the stage of terminal chondrocyte differentiation and is undesired in cartilage regenerative approaches as apoptosis follows and formation of abnormal ECM components and mineralization of the tissue can occur [2]. This resembles processes within the growth plate, where the interplay of several factors, including BMPs, IHH and others, mediates the replacement of cartilage by bone via endochondral ossification. This involves chondrocyte maturation, hypertrophy and subsequent apoptosis, while osteoprogenitor cells differentiate into osteoblasts and replace the cartilage with mineralized bone tissue [9, 10]. This observation corresponds to the related in vivo data, where MSCs and *BMP2* induced tissue hypertrophy and osteophyte formation, when transplanted at orthotopic [11, 12] or ectopic [13, 14] sites.

The transcription factor sex-determining region Y-type high-mobility-group-box (SOX) 9 (encoded by *SOX9*), has been identified as one capable of chondrogenic induction, while inhibiting hypertrophic stages in primary MSCs [15–17]. Known as a master regulator of chondrogenesis in embryonic cells, SOX9 is needed for chondrogenic mesenchymal condensation in embryonic limb formation [18], and the DNA binding domains of SOX9 directly control expression of several chondrogenic marker proteins, e.g. collagen (COL) type IIa1, COL type IIa2, and aggrecan, among others. It is expressed in all chondroprogenitor cells and chondrocytes, but not in hypertrophic chondrocytes [17, 19].

As SOX9 is a transcription factor it can only be delivered efficiently via genetic approaches. The aim of the present study was to explore the effects of adenoviral delivery of *SOX9* compared to *TGFB1*, or *IGF1* cDNA or combinations thereof, on chondrogenesis of primary MSCs and to investigate whether the levels and extent of chondrogenic hypertrophy are influenced by the choice of the transgene in an established in vitro aggregate culture model.

### Gene therapy for cartilage regeneration

Gene therapy involves the transfer of genes, or more usually cDNAs, to target cells that will express the transgene. This is accomplished using viral or non-viral vectors that can deliver the transgene to the nucleus of the recipient cell. Gene transfer using non-viral vectors is known as transfection.

Non-viral vectors can be as simple as DNA plasmids, but transfection with plasmids is inefficient. Transfection efficiency can be improved by combining the plasmid with certain types of nanoparticles, by formulating them with a matrix to produce a “gene activated matrix” (GAM) or by engaging a physical stimulus as in electroporation, sonoporation or magnetofection [20]. Even when augmented in these ways, transfection efficiency is usually too low to be useful for cartilage repair as presently envisioned, where the goal is to express chondrogenic morphogens or transcription factors. For this reason, most progress has been made using viral vectors.

Viral vectors exploit the high natural infectivity of viruses [21]. In engineering viruses as vectors, the aim is to remove components of the viral genome that contribute to pathogenicity and replace them with therapeutic cDNAs. Although viral vectors are much more efficient than non-viral vectors, they are more difficult to produce and, when considering human application, they raise more safety issues. The recombinant viruses most commonly used in human gene therapy trials are based upon retroviruses, lentiviruses, adenovirus, and adeno-associated virus (AAV). Recent marketing approvals from the EMA and FDA have gone to gene therapeutics using lentivirus and AAV. In the studies reported here we have used adenovirus because it is highly efficient, straightforward to produce in the laboratory at high titre and typically expresses for around 2–3 weeks, which may be ideal for initiating a sustained regenerative response.

The use of gene transfer to stimulate cartilage repair was first suggested 25 years ago by Evans and Robbins [22]. Since then, a large literature has accumulated with pre-clinical animal models to demonstrate proof of principle using a variety of vectors delivering chondrogenic cDNAs by in vivo and ex vivo strategies [23, 24]. These studies have coincided with the emergence of mesenchymal stromal cells (MSCs) as clinically relevant agents of cartilage repair, and investigations into the use of genetically modified MSCs to regenerate cartilage are popular [25]. Most of these studies have used cDNAs encoding morphogens such as TGF-β, BMPs-2, − 7 or − 9, and IGF-1 and, while giving initially favourable results ultimately produce a regenerate that undergoes endochondral ossification. In response to this, the present study uses a construct expressing *SOX9* which may not provoke this problem [26–30].

## Methods

### Recombinant adenoviral vectors

The adenoviral vectors for *TGFB1*, *IGF1*, firefly luciferase (*LUC*) and green fluorescent protein (*GFP*) were generated by *cre*-*lox* recombination as described earlier [31, 32]. The first generation adenoviral vector, serotype 5, carrying a human *SOX9* - *GFP* fusion cDNA (*SOX9/GFP*) was generated using the Ad. Easy system as earlier described [33]. The resulting vectors were designated Ad.*SOX9*, Ad.*TGFB1*, Ad.*IGF1*, Ad.*LUC*, or Ad.*GFP*. The suspensions of recombinant adenovirus were prepared by amplification in 293 cells. After this, the suspensions were purified by three consecutive CsCl gradients [31]. Optical densitometry at 260 nm and standard plaque assay were used to estimate the viral titers, which ranged between 10^12^ and 10^13^ particles/mL.

### Cultivation of bone marrow-derived MSCs, adenoviral transduction and aggregate culture

Bone marrow was received from the proximal femurs of 10 patients, aged 36–65 years (mean age 53), undergoing total hip arthroplasty. The underlying pathology was primary osteoarthritis in all cases and informed written consent was obtained from all volunteers as approved by the institutional review board of the University of Würzburg that agreed to the entire study protocol (number of the approval 82/08). MSC isolation and culture were performed as described earlier [7, 8]. The culture medium for amplification consisted of DME/F-12 medium (containing 10% FBS and 1% penicillin/streptomycin), and cells were plated at 2–3 × 10^8^ nucleated cells per 150 cm^2^ flask (Falcon, Beckton Dickinson Lab ware, Franklin Lakes, NJ). After 3 days unattached cells were removed, and adherent colonies were cultured at 37 °C, in a humidified atmosphere of 95% air and 5% CO_2_ in DME/F-12 medium with 10% FBS. Changes of the medium were performed every 3–4 days. At the time of confluence (approximately 1.2 × 10^6^ cells/150 cm^2^ flask), the cultures were washed with phosphate buffered saline (PBS). Then the cultures were infected in 750 μL serum-free DMEM for 2 h at a dose of 5 × 10^2^ infectious particles (ip)/cell of Ad.*SOX9*, Ad.*TGFB1*, Ad.*IGF1*, alone or in combination at 5 × 10^2^ ip/cell for each vector as described in the respective experiments later. Control groups were infected with similar doses of Ad.*GFP*, Ad.*LUC*, or remained uninfected and were maintained in the presence or absence of recombinant human TGF-β1 protein at 10 ng/mL (R&D Systems, Minneapolis, MN, USA). After two hours of viral infection, the supernatant was aspirated and replaced with complete DME/F-12 medium.

The day after infection, MSCs were detached with trypsin (0.05% trypsin-EDTA (Invitrogen), washed and then placed in pellet culture as described previously [7, 8]. MSCs were suspended to a concentration of 1 × 10^6^ cell/mL in serum-free DMEM containing 1 mM pyruvate, 1% ITS+ Premix, 37.5 μg/mL ascorbate-2-phosphate and 10^− 7^ M dexamethasone (all Sigma, St. Louis, MO), and aliquots of 3 × 10^5^ cells were transferred to polypropylene conical 15-mL-tubes (Greiner BioOne Int. AG, Kremsmuenster, Austria) and spun in order to induce aggregate formation. Uninfected controls were also maintained in the presence or absence of 10 ng/mL recombinant human (rh) TGF-β1 protein (R&D Systems, Minneapolis, MN, USA). Pellets were cultured at 37 °C, and medium changes were performed every 2–3 days. In addition, rhTGF-β1 was also freshly added to the appropriate cultures. Pellets were harvested at various defined time points for further analyses.

### Transgene expression analyses

Green fluorescent cells in monolayer and aggregate culture following transduction were identified by fluorescence microscopy. To quantitatively confirm transduction efficiencies vectors encoding *SOX9/GFP* or *GFP* alone we employed fluorescence and light microscopy on five representative high power fields of each of three aggregate midsections for three aggregates per group and time point and quantified the number of green cells relative to the total number of cells. As *SOX9/GFP* is expressed as a fusion construct, GFP+ cells identify *SOX9* expressing cells in the *SOX9/GFP* group, and allow assessment of the extent and duration of *SOX9* transgene expression.

At day 3, 7, and 14 cell lysates of the transduced MSCs in the aggregate culture were collected, frozen at − 80 °C and analyzed for SOX9 protein production by western blot as described previously [32]. The protein content of each lysate was determined using the Rotiquant according to the instructions of the supplier (Carl Roth GmbH, Karlsruhe, Germany). Twenty μg of protein were boiled for 5 min in SDS-polyacrylamide gel electrophoresis buffer (10 mM Tris, pH 6.8; 7.5% glycerol, 10% SDS, 0.025% bromphenol blue). Thereafter, proteins were separated by 12% SDS-PAGE and electrotransferred to nitrocellulose membranes. To inhibit non-specific binding, the membranes were treated with buffer containing 0.1% Tween 20, 2% horse serum, 2.5% bovine serum albumin (BSA), and 2.5% milk powder in PBS for 2 h. Then, the membranes were incubated overnight at 4 °C in 0.1% Tween 20, 1% horse serum and 1% milk powder in PBS with the primary human antibody anti-SOX9 (1:100; Acris Antibodies GmbH, Hiddenhausen, Germany). After 3 wash steps in washing solution (10 mM Tris, pH 7.5, 140 mM NaCl, 2 mM EDTA, 0.1 Triton X-100, 1% horse serum, 1% BSA, and 1% milk powder), the membrane was incubated for 1 h with horseradish peroxidase-conjugated anti-rabbit IgG (1:2000; Sigma-Aldrich) using a solution containing in 0.1% Tween 20, 1% horse serum, 1% BSA, and 1% milk powder in PBS. After further washes, signals were detected by chemiluminescence using the ECL system (Amersham Biosciences, GE Healthcare Life Sciences, Freiburg, Germany).

Media conditioned by the respective aggregates over a 24-h period were collected at day 3, 7, 14 and 21 of culture and assayed for TGF-β1 or IGF-1 protein production using the appropriate commercially available ELISA kits as directed by the supplier (R&D Systems).

### Histology and immunohistochemistry

For histology, aggregates were fixed in 4% paraformaldehyde for 1 h, followed by dehydration in graded alcohols, paraffin embedding, sectioning to 4 μm, and staining with haematoxylin/eosin (H&E) or alcian blue (Sigma) as described previously [7, 8]. For visualisation of ALP activity, a histochemical assay was performed according to the instructions of the supplier (Sigma).

Immunohistochemistry on alternate sections was performed as described previously [7]. Briefly, following the respective pre-treatments with pepsin (1 mg/mL), or chondroitinase ABC (Sigma; 5 U/mL), or trypsin (0.25%) sections were incubated overnight with the following primary antibodies: monoclonal anti-COL type II (Acris Antibodies GmbH, Hiddenhausen, Germany), anti-chondroitin-4-sulphate (CS4) (Millipore GmbH, Schwalbach, Germany) or anti-collagen type X (COL type X) (Calbiochem, Bad Soden, Germany). Immunohistodetection was performed by treatment with Advance™ HRP link and Advance™ HRP enzyme (Dako, Hamburg, Germany) followed by diaminobenzidine staining (DAB kit; Sigma), and slides were finally counterstained with hemalaun (Merck, Darmstadt, Germany). In addition, controls with non-immune Ig G (Sigma) instead of the primary antibodies were also performed.

### Annexin 5 assay

As a marker of hypertrophy and apoptosis annexin 5 expression in the cultures was determined as directed by the supplier (APOAC; Sigma) and previously described [7, 8]. In brief, the test uses a double labelling with the red fluorochome Cy3.18/Ann5-Cy3 that binds to early apoptotic cells and 6-carboxyfluorescein diacetate (CFDA; non-fluorescent), which is converted to 6-carboxyfluorescein (green fluorescent) by living cells. After 10 or 21 days of culture, aggregates were washed with PBS twice and incubated with double labelling staining solution for 10 min, before they were washed again and fixed in 4% paraformaldehyde before tissue processing to 4 μm paraffin sections. Assessment of living and apoptotic cells was performed on representative sections by using a fluorescence microscope with the appropriate green and red filters.

### Biochemical assays

For analysis of cell proliferation in aggregates the CellTiter-Glo® Luminescent Cell Viability Assay was performed as directed by the supplier (Promega) and as described earlier [7, 8]. Briefly, for quantitative detection of adenosine 5′-triphosphate (ATP), which correlates with the number of viable cells, pellets were disrupted using a pellet pestle, mixed with 100 μL of CellTiter-Glo® reagent, and luminescence was measured after 10 min using a plate-reading luminometer.

For quantitative assessment of glycosaminoglycan (GAG) content, pellets were digested with papain solution (1 μg/mL, Sigma), and total GAG content was measured by reaction with 1,9-dimethylmethylene blue (DMMB) using the Blyscan™ Sulfated Glycosaminoglycan Assay (Biocolor Ltd., Newtownabbey, Northern Ireland) as directed by the manufacturer. DNA content of aggregates was also assessed for normalisation, using the Quant-iT™ PicoGreen® kit as indicated by the supplier (Invitrogen GmbH, Karlsruhe, Germany).

Alkaline phosphatase (ALP) activity was measured densitometrically at 405 nm as described previously [7, 8]. Briefly, pellets were dispersed mechanically followed by supplementation with 0.1 mL of alkaline lysis buffer for 1 h (0.1 M glycin, 1% triton X-100, 1 mM MgCl_2_, 1 mM ZnCl_2_), 0.1 mL of lysis buffer with p-nitrophenylphosphate (2 mg/mL; Sigma) for 15 min, followed by 50 μL 50 mM NaOH stop solution. Optical densities were determined at 405 nm in an ELISA reader. Relative ALP activities were determined using a standard curve made from p-nitrophenol (Sigma), and normalized to the DNA content.

### Gene expression analyses

Total RNA was extracted from MSC aggregates at days 3, 7, 14 and 21. 6–10 pellets per group and time point were pooled and homogenised using a pellet pestle and repeated tituration in 3.5 μl β-mercaptoethanol and 350 μl lysis buffer (Invitrogen). Extraction of total RNA was subsequently performed by using separation columns (NucleoSpin RNA II kit; Macherey-Nagel GmbH, Düren, Germany) with a DNase treatment step according to the manufacturer’s instructions. For random hexamer primed cDNA synthesis RNA from aggregates of each condition (2 μg each group) was used utilizing BioScript reverse transcriptase (Bioline GmbH, Luckenwalde, Germany).

Real-time quantitative PCR analyses were performed for a more accurate assessment of mRNA expressions levels of chondrogenic and hypertrophy marker genes as described previously [7, 8]. The annealing temperatures, sequences and product sizes of forward and reverse primers used for the following genes (HUGO gene symbol): collagen type II alpha 1 (*COL2A1*), aggrecan (*ACAN*), *SOX9*, collagen type X alpha 1 (*COL10A1*), alkaline phosphatase (*ALPL*), are listed in Table 1. Elongation factor 1α (*EEF1A1*) served as internal control and housekeeping gene. Briefly, one microliter of each cDNA was used as template for amplification in a 50 μL reaction volume using BioTaq DNA Polymerase Taq (Bioline GmbH) and 50 pmol of gene-specific primers and conditions as listed in Table 1. Real-time PCR was performed with the DNA Engine Opticon system (MJ Research, Waltham, MA) and SYBR Green (Biozym Scientific GmbH, Hessisch Oldendorf, Germany) was used as fluorescent dye. Amplicon specificities were finally confirmed by melting curve analyses by gel electrophoresis of test PCR reactions. Quantification of mRNAs was performed using the ΔΔCT method normalised to the expression levels of the housekeeping gene *EEF1A1* and relative to values from the control group as described previously [7, 8]. Each PCR was performed in triplicate on three separate marrow preparations for each independent experiment.

**Table 1:** Primer sequences and product sizes for real time RT-PCR

Gene	RT-PCR primer sequences (5′-3′)	Annealing temp. (°C)	Product size (bp)
**Chondrogenic marker genes**			
*COL2A1*	Sense: TTTCCCAGGTCAAGATGGTC Antisense: CTTCAGCACCTGTC CACCA	58	374
*SOX9*	Sense: AGTACCCGCACTTGCACAAC Antisense: CGTTCTTCACCGACTTCCTC	58	263
*ACAN*	Sense: TCGAGGACAGCGAGGCC Antisense: TCGAGGGTGTAGCGTGTAGAGA	54	392
**Hypertrophy and osteogenic marker genes**			
*COL10A1*	Sense: CCCAACACCAAGACACAGTTC Antisense: GACTTCCGTAGCCTGGTTTTC	54	468
*ALPL*	Sense: GGAACTCCTGACCCTTGACC Antisense: CCACCATCTCGGAGAGTGAC	51	454
**Internal control**			
*EEF1A1*	Sense: TGCCCCTCCAGGATGTCTAC Antisense: CACGGCCCACAGGTACTG	60	59

### Statistical analysis

The numerical data from the ELISA, DNA, GAG, ATP, and ALP content, as well as the real-time quantitative RT-PCR analyses were expressed as mean values **±** standard deviation (SD). Each experiment was performed in triplicate or quadruplicate *(N =* 3–4) and repeated on at least 3 and up to 6 individual bone marrow preparations from several different patients *(N =* 3–6). Numerical data on protein level were subjected to variance analysis (one or two factor ANOVA). Statistical significance was determined by student’s t-testing. Level of *p* < 0.05 was considered significant.

## Results

### Transgene expression by genetically modified MSCs in pellet culture

**Fig. 1:**
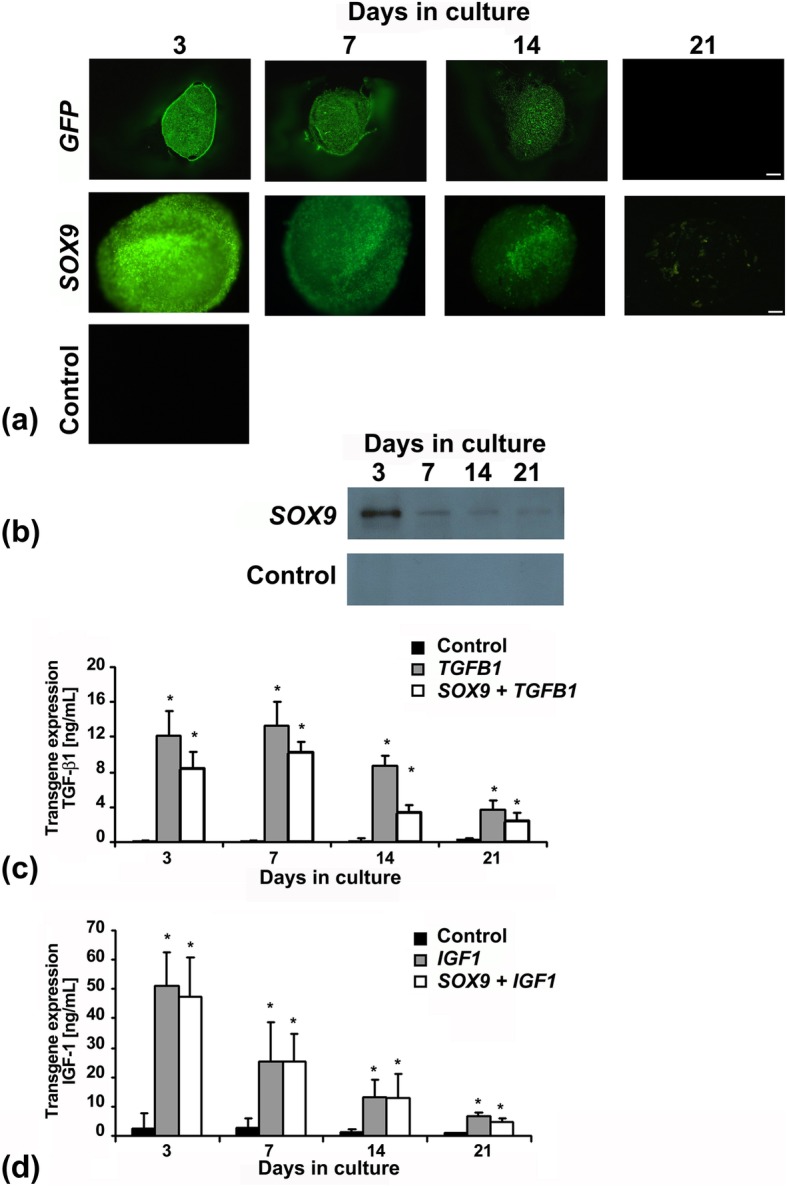
Transgene expression by MSCs during 21 days of aggregate culture following adenoviral gene transfer with *GFP*, *SOX9*, *TGFB1* or *IGF1* alone or in combination. Primary MSCs were infected with Ad.*GFP*, Ad.*SOX9*, Ad.*TGFB1*, Ad.*IGF1* alone or in combinations at 5 × 10^2^ vp/cell, seeded into aggregates and analysed for respective transgene expression during a 3 week time course. (**a**) *GFP* transgene expression was detected by fluorescence microscopy, panels are reproduced at low magnification (50x; bar = 200 μm) as indicated (**b**) Production of SOX9 protein was analysed by Western blot of cell lysates. Representative gels of experiments from three independent marrow preparations are shown; lysates from 10 aggregates per time point represent one band of the gel. (**c**) *TGFB1* transgene expression was evaluated by measurement of TGF-β1 protein concentration in the conditioned media of the respective aggregate cultures over a 24-h period at days 3, 7, 14 and 21 compared to Ad.*GFP* controls. The data represent mean values ± SD from measurements of supernatants of *n* = 3 aggregates per condition and time point; *n* = 3 marrow preparations. Asterisks indicate values that are statistically different (*p* < 0.05) from marker gene–transduced control cultures or between samples as indicated. (**d**) *IGF1* transgene expression was evaluated by quantification of IGF-1 protein concentration in the conditioned media of the respective aggregate cultures over a 24-h period at days 3, 7, 14 and 21 compared to Ad.*GFP* controls. The data represent mean values ± SD from measurements of supernatants of *n* = 3 aggregates per condition and time point; *n* = 3 marrow preparations. Asterisks indicate values that are statistically different (*p* < 0.05) from marker gene–transduced control cultures

Following transduction with Ad.*GFP* or Ad.*SOX9* vector and placement into pellet culture, expression of the *GFP* or *SOX/GFP* transgene was observed by fluorescence microscopy. This showed initially high levels of green fluorescence until day 7 of culture and gradually declining levels of green fluorescence thereafter toward almost background levels by day 21 (Fig. 1a). Untransduced, Ad*.TGFB1* or Ad.*IGF1* transduced cultures were also maintained and served as controls which were not green fluorescent (Fig. 1a). Quantification of the transduction efficiencies revealed that > 95% of GFP+ cells were seen at day three in the *SOX9/GFP* and *GFP* groups respectively, confirming high levels of *SOX9* and *GFP* transgene expression using first generation adenoviral vectors. Thereafter the transgene expression levels declined at days 7, 14 and 21 as reflected by decreasing ratios of GFP+ cells in the respective *SOX/GFP* (75, 45, and 16%), or *GFP* (78, 56, and 13%) groups.

Expression of the *SOX9* transgene was further investigated by western blot analyses from lysates of Ad.*SOX9* modified pellet cultures, compared to Ad.*GFP* controls, showing high levels of *SOX9* expression at day 3 of culture and subsequently declining levels during the time course, with the GFP controls revealing no visible bands at the same time points (Fig. 1b).

Cultures which were transduced with Ad.*TGFB1* alone (*TGFB1*) or together with Ad.*SOX9* (*SOX9 + TGFB1*) were analysed for 24-h accumulation of TGF-β1 protein in the conditioned media using ELISA, with *GFP* modified cultures serving as negative controls (Fig. 1c). While *GFP* control cultures showed only background levels of expression, high levels of TGF-β1 protein production were reached by the *TGFB1* and the *SOX9* + *TGFB1* cultures at day 3 of culture with declining levels thereafter during the 21 day time-course (Fig. 1c). Similarly, expression of the *IGF1* transgene was investigated in the cultures infected with Ad.*IGF1* alone (*IGF1*) or together with Ad.*SOX9* (*SOX9* + *IGF1*). These were analysed for 24-h accumulation of IGF-1 protein in the conditioned media using ELISA, with GFP modified cultures serving as negative controls (Fig. 1d). High levels of IGF-1 protein production were reached by both *IGF1* modified cultures (*IGF1* and *SOX9 + IGF1*) with values of approximately 40–60 ng/mL at day 3, and 20–35 ng/mL at day 7 of culture with declining levels thereafter (Fig. 1d). Levels of IGF-1 protein in media conditioned by Ad.*GFP* infected cultures were low (Fig. 1d), equivalent to the levels observed in the naïve controls (data not shown).

### Histology and immunohistochemistry of the chondrogenic phenotype

**Fig. 2:**
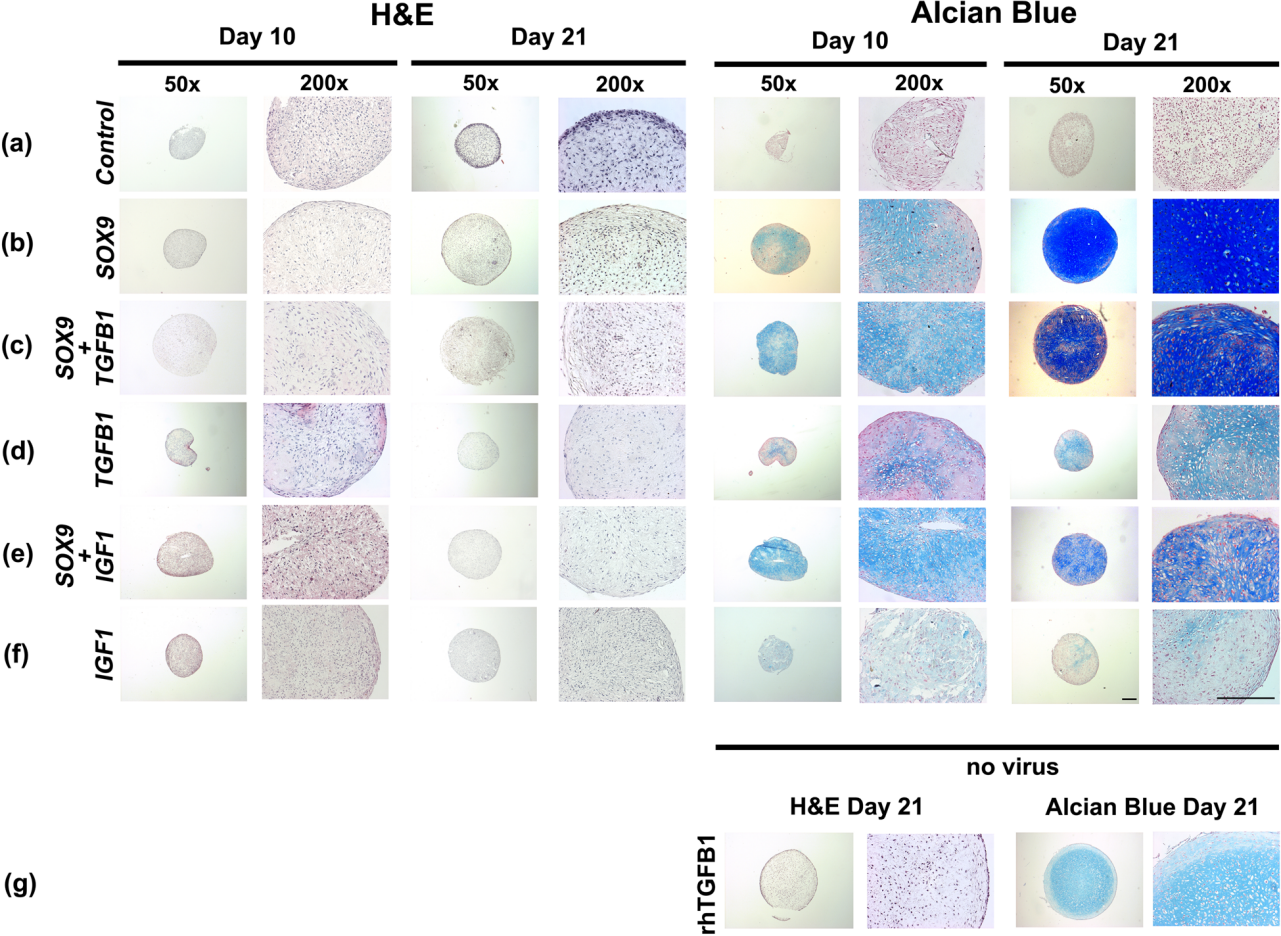
Histological appearance of MSC pellets after chondrogenic induction by adenoviral gene transfer of *SOX9* compared to *TGFB1* or *IGF1* alone or in combination. MSC monolayer cultures were infected with Ad.*GFP* (Control; **a**), Ad.*SOX9* (**b**), Ad.*TGFB1* + Ad.S*OX9* (**c**), Ad.*TGFB1* alone (**d**), Ad.*IGF1* + Ad.*SOX9* (**e**), or Ad.*IGF1* alone (**f**) at 5 × 10^2^ vp/cell, seeded into aggregates 24 h after infection and cultured in serum-free medium for 21 days. Representative sections after 10 and 21 days are shown, which were stained with H & E for evaluation of cellularity and cell morphology (left panels), and with alcian blue (right panels) for detection of matrix proteoglycan. Uninfected, with recombinant TGF-β1 treated pellets after 21 days of culture are shown for comparison (**g**). Panels (**a-g**) are reproduced at low (50x; bar = 200 μm) or high (200x; bar = 50 or 100 μm) magnification as indicated

Cells transduced with *GFP* were not chondrogenic (Fig. 2a), but genetic modification of MSCs with adenoviral vectors encoding *SOX9* induced large pellets with abundant proteoglycan accumulation (Fig. 2b). MSCs transduced with *TGFB1* (Fig. 2d) displayed a moderate chondrogenic phenotype*,* but *IGF1* alone was not chondrogenic (Fig. 2f). When combined with *TGFB1* or *IGF1,* the chondrogenic response to *SOX9* was weaker (Fig. 2c,e).

**Fig. 3:**
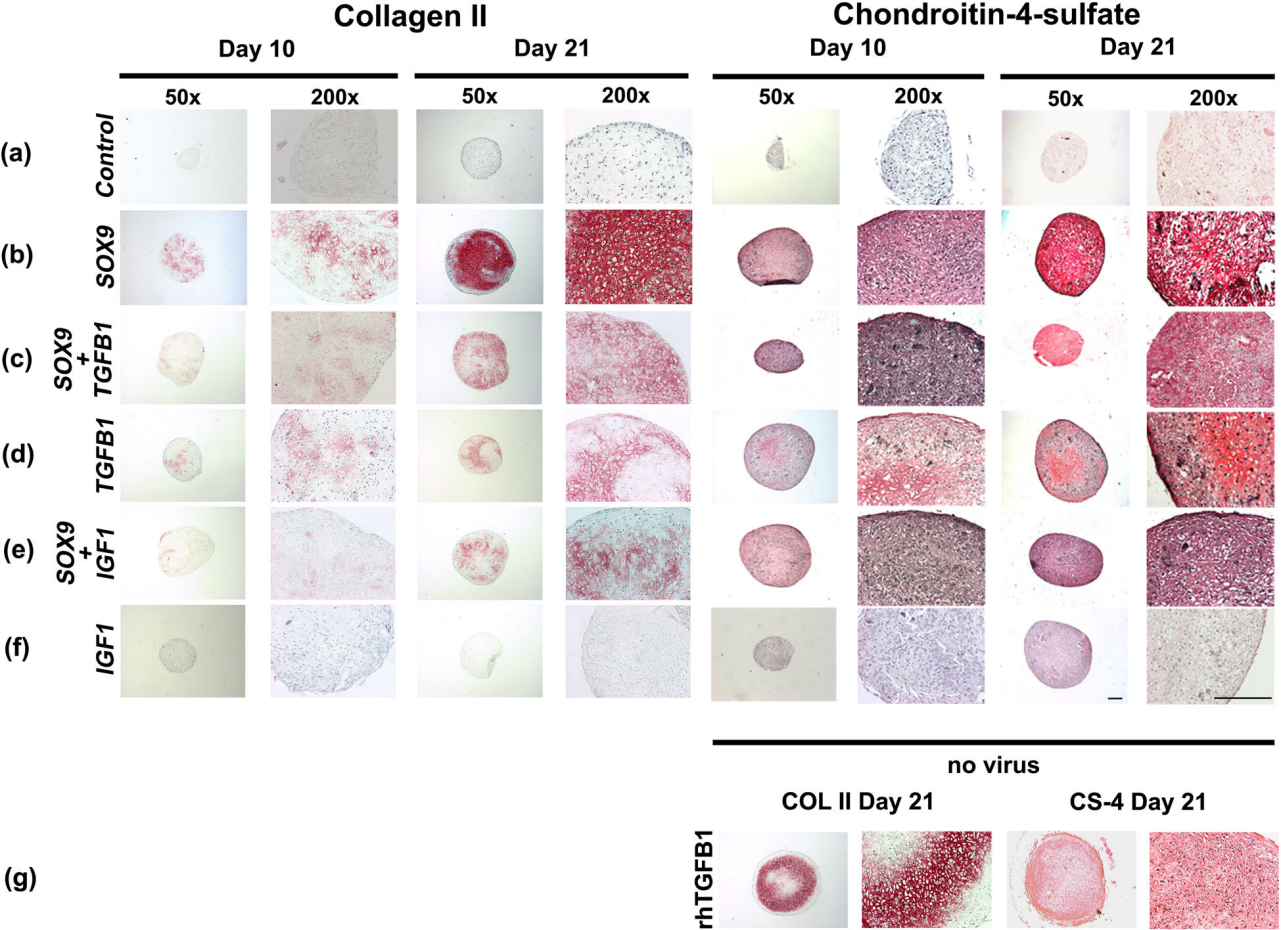
Immunohistochemical analyses for cartilage matrix proteins in MSC pellets after chondrogenic induction with adenoviral gene transfer of *SOX9* compared to *TGFB1* or *IGF1* alone, or in combination. Monolayer cultures of MSCs were infected with Ad.*GFP* (Control; **a**), Ad.*SOX9* (**b**), Ad.*TGFB1* + Ad.*SOX9* (**c**), Ad.*TGFB1* alone (**d**), Ad.*IGF1* + Ad.*SOX9* (**e**), or Ad.*IGF1* alone (**f**) at 5 × 10^2^ vp/cell, seeded into aggregates 24 h after infection and cultured in serum-free medium for 21 days. Immunohistochemical staining was performed on representative sections after 10 and 21 for collagen type II (COL II; left panels), the predominant collagen hyaline cartilage, and chondroitin-4-sulphate (CS4; right panels) one of the proteoglycan matrix components (**a-f**). Uninfected, with recombinant TGF-β1 treated pellets after 21 days of culture are shown for comparison (**g**). Panels (**a-g**) are reproduced at low (50x; bar = 200 μm) or high (200x; bar = 50 or 100 μm) magnification as indicated, and regions of positive immunostaining appear red

Immunohistochemistry for COL type II, the main collagen of cartilage, and CS4, one of the small monomers of the polysaccharide fraction of cartilage GAGs, confirmed the results of H&E and alcian blue staining. Enhanced production of these cartilage matrix components at days 10 and 21 of culture was detected in the aggregate groups *SOX9*, *TGFB1*, *SOX9* + *TGFB1* and *SOX9* + *IGF1* (Fig. 3b-e), relative to the *GFP* (Fig. 3a) and *IGF1* alone groups (Fig. 3f), which were not chondrogenic.

Uninfected aggregates maintained in the presence or absence of recombinant human (rh) TGF-β1 (10 ng/mL) or IGF-1 (50 ng/mL) protein revealed a chondrogenic phenotype in the rhTGF-β1 group (Figs. 2 and 3g) but not the rhIGF-1 group (data not shown); control cultures lacking growth factor supplementation were non-chondrogenic (data not shown).

### Hypertrophic differentiation and apoptosis

**Fig. 4:**
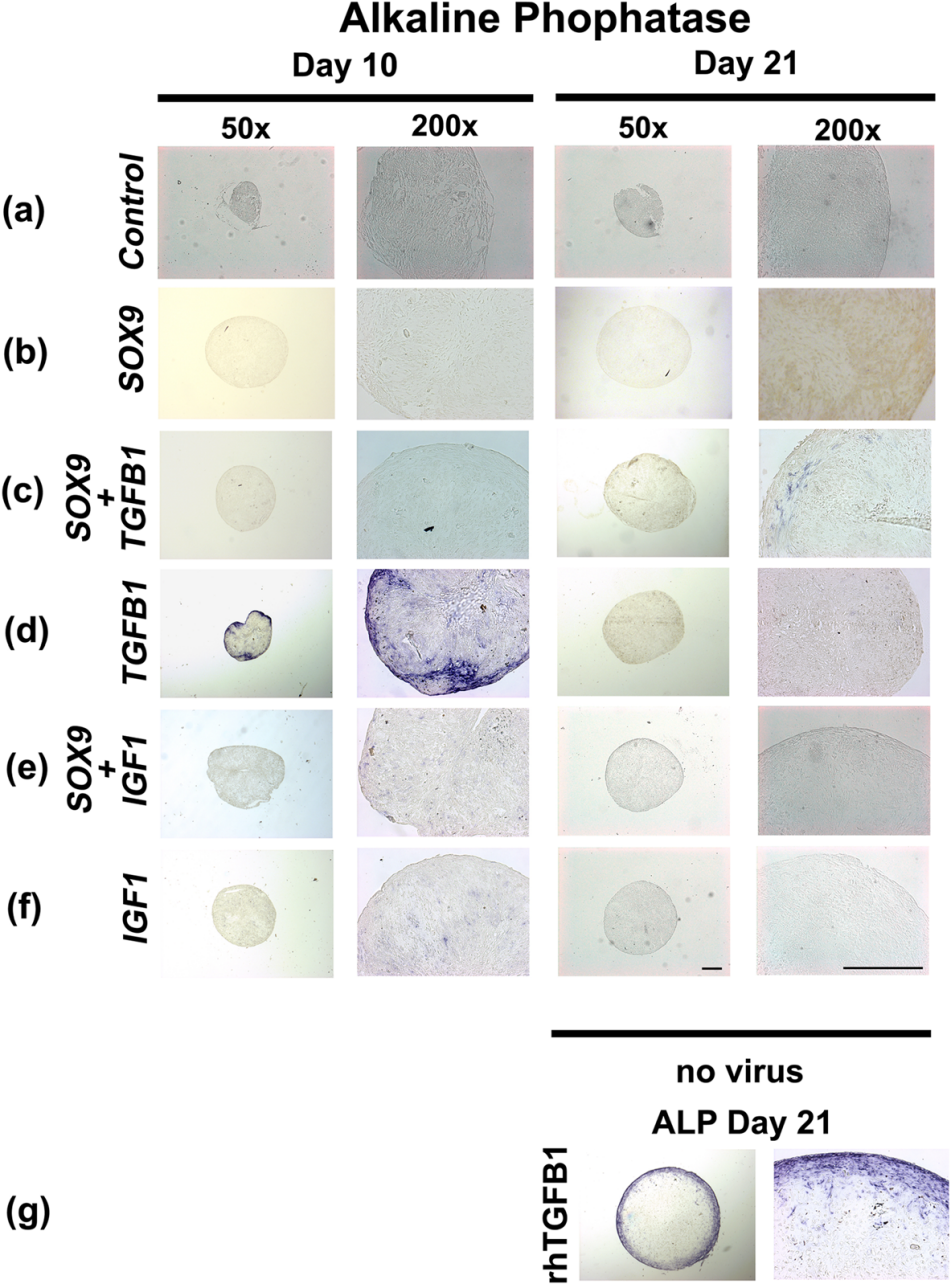
Histochemical analyses for hypertrophy of MSC pellet cultures after chondrogenic induction with adenoviral gene transfer of *SOX9* compared to *TGFB1* or *IGF1* alone, or in combination. MSC pellet cultures genetically modified with Ad.*GFP* (Control; **a**), Ad.*SOX9* (**b**), Ad.*TGFB1* + Ad.*SOX9* (**c**), Ad.*TGFB1* alone (**d**), Ad.*IGF1* + Ad.*SOX9* (**e**), or Ad.*IGF1* (**f**) were maintained in serum-free medium for 21 days. Representative sections after 10 and 21 days are shown, which were stained histochemically for alkaline phosphatase (ALP). Uninfected, with recombinant TGF-β1 treated pellets after 21 days of culture are shown for comparison (**g**). (**a-g**) Panels are reproduced at low (50x; bar = 200 μm) or high (200x; bar = 50 or 100 μm) magnification as indicated, and regions of positive staining for ALP appear blue

Histochemical staining for ALP and immunohistochemical analysis for COL type X (data not shown) were used for evaluating chondrocyte hypertrophy (Fig. 4). In the ALP staining no or only very weak positive staining could be observed in the *GFP* control pellet cultures (Fig. 4a), as well as the non-chondrogenic *IGF1* (Fig. 4f), and the chondrogenic *SOX9* (Fig. 4b), and *SOX9* + *IGF1* (Fig. 4e) groups. In contrast, aggregates transduced with Ad.*TGFB1* alone showed abundant blue staining for ALP especially in the area of the outer rim of the aggregates (Fig. 4d). This was also strongly present in the rhTGF-β1 group (Fig. 4g). Cultures transduced with Ad.*SOX9* together with Ad.*TGFB1* (Fig. 4c) revealed weaker ALP staining compared to the Ad.*TGFB1* alone group with primarily pericellular staining pattern within the aggregates (Fig. 4d).

**Fig. 5:**
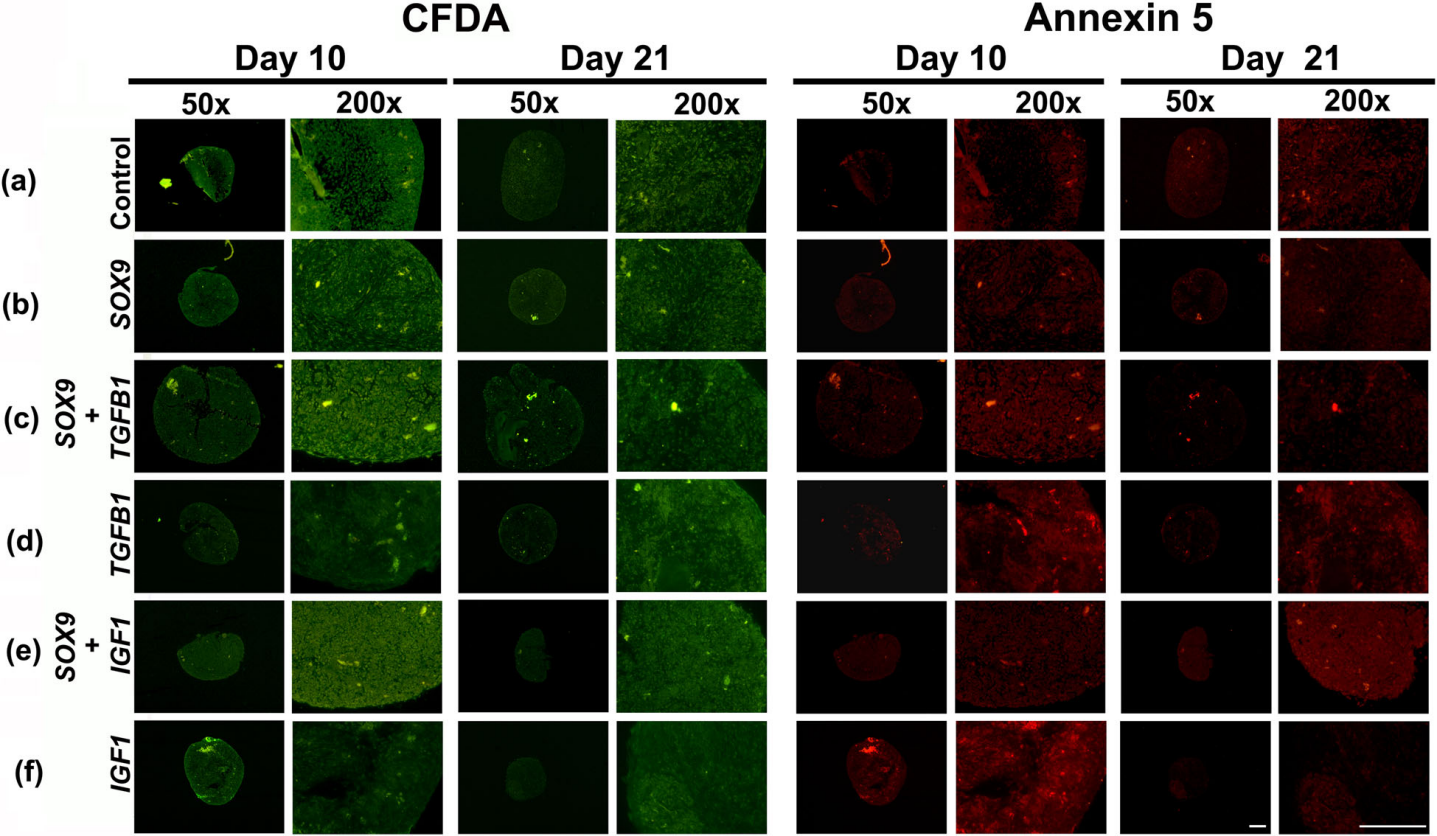
Analysis for cell viability and apoptosis within MSC pellets after chondrogenic induction with adenoviral *SOX9*, *TGFB1* or *IGF1* gene transfer alone or in combination. Following genetic modification with Ad.*GFP* (Controls; **a**) Ad.*SOX9* (**b**), Ad.*SOX9* + Ad.*TGFB1* (**c**), Ad.*TGFB1* (**d**), Ad.*SOX9* + Ad.*IGF1* (**e**) or Ad.*IGF1* (**f**) and at 5 × 10^2^ vp/cell and aggregate culture. Pellets were double-stained with CFDA (left panels) and annexin 5 (right panels) at day 10 and 21 of culture. Fluorescence microscopy images of representative sections are shown. Note that living cells are stained green with CFDA, late apoptotic cells red with annexin 5-Cy3, while early apoptotic cells stained for both CFDA and Annexin 5. Panels are reproduced at low (50x; bar = 200 μm) or high (200x; bar = 50 μm) magnification as indicated

Pellet cultures were probed using double fluorescence staining with Ann5-Cy3/6-CFDA to identify living and apoptotic cells (Fig. 5) in untransduced controls and following adenoviral gene transfer of the various gene combinations. In all groups high levels of green fluorescence (viable cells) could be detected after 10 and 21 days of culture (Fig. 5, left panels) without major differences between groups (Fig. 5 a-f). Staining for apoptosis with annexin 5 (Fig. 5; right panels) detected only very few annexin 5 positive cells (red fluorescent) in the control group (a), as well as the *SOX9* group (b), while many annexin 5 positive cells were found in all other groups at days 10 and 21 of culture (c-f).

Similar patterns of hypertrophy and apoptosis were observed in the untransduced control aggregates that were maintained in the presence or absence of human recombinant IGF-1 or TGF-β1 protein (data not shown).

### Biochemical assays - cell proliferation, GAG content and ALP activity

**Fig. 6:**
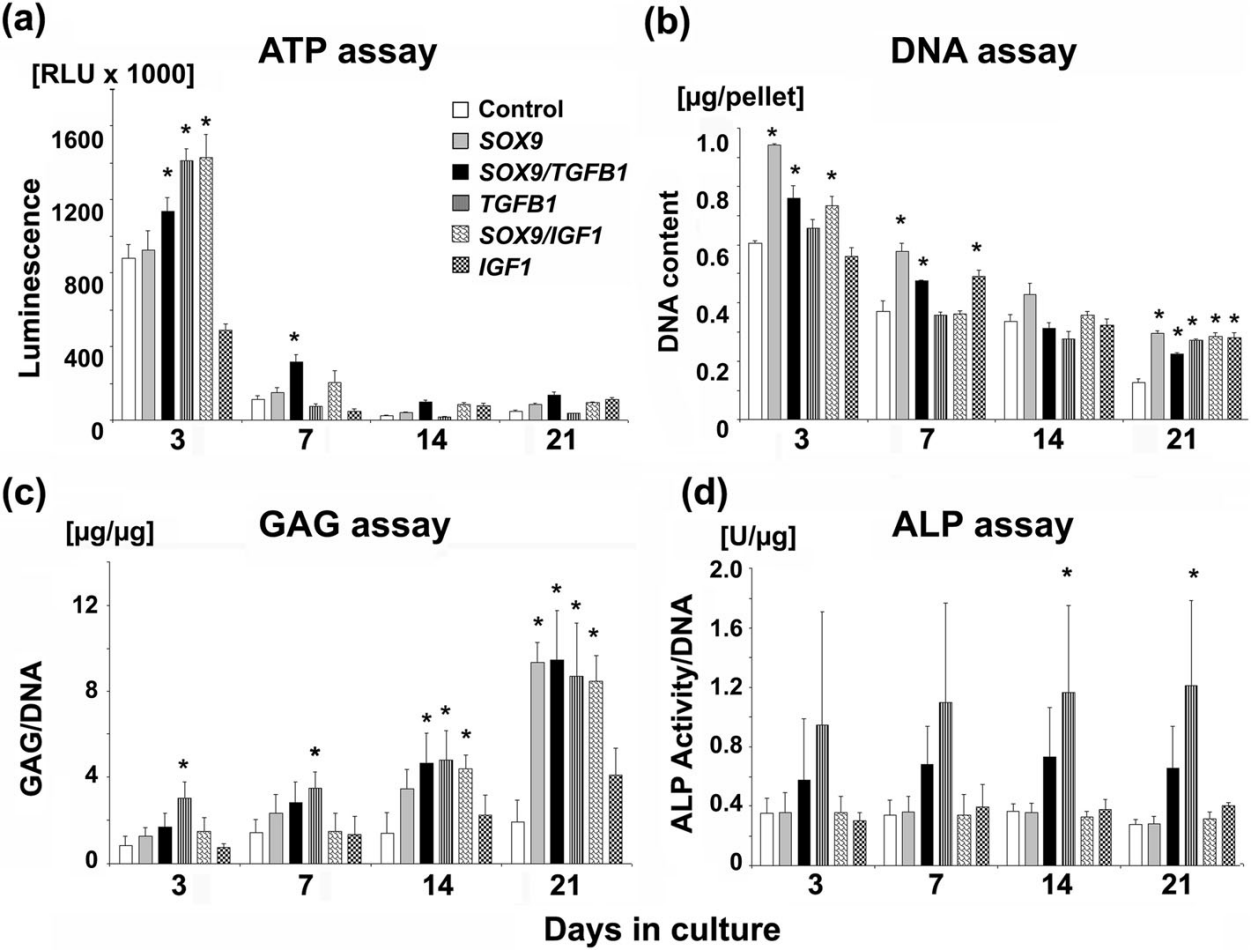
Biochemical composition of MSCs during three weeks of pellet culture following adenoviral gene transfer with *GFP*, *SOX9*, *TGFB1* or *IGF1* alone or in combination. Primary MSCs were infected with Ad*.GFP* (Control), Ad.*SOX9* (*SOX9*), Ad.*TGFB1* + Ad.*SOX9* (*SOX9* + *TGFB1*), Ad.*TGFB1* alone (*TGFB1*), Ad.*IGF1* + Ad.*SOX9* (*SOX9* + *IGF1*), or Ad.*IGF1* alone (*IGF1*) at 5 × 10^2^ vp/cell, seeded into aggregate cultures and maintained in serum-free medium for 21 days. At days 3, 7, 14 and 21 biochemical assays were performed to measure (**a**) cell proliferation by the ATP assay, (**b**) DNA content, (**c**) GAG synthesis via GAG/DNA ratios, and (**d**) ALP activity normalized to the DNA content. (**a-d**) The data represent mean values ± SD from *n* = 3 aggregates per condition and time point from marrow preparations of *n* = 3 different patients. Asterisks indicate values that are statistically different (*p* < 0.05) from marker gene vector–transduced control cultures or between samples

At day 3 of culture the cell proliferation rate was high in all groups of MSC aggregates, with highest rates being observed in the chondrogenic groups *SOX9*, *SOX9* and *TGFB1*, *TGFB1* alone and *SOX9* and *IGF1* compared to the non-chondrogenic *GFP* controls and the *IGF1* alone group (Fig. 6a). Thereafter, the rate of proliferation decreased in all groups tested with highest values for the *SOX9* + *TGFB1* and *SOX9* + *IGF1* groups at day 7 and without major differences among groups at days 14 and 21 of culture (Fig. 6a).

A similar pattern was observed using the DNA assay, where high values were observed at day 3 in all groups, with highest values being present in the chondrogenic groups *SOX9*, *SOX9* + *TGFB1*, *TGFB1* alone and *SOX9* + *IGF1* compared to the non-chondrogenic *GFP* control and the *IGF1* alone group (Fig. 6b). Thereafter, the DNA content decreased in all groups tested without clear differences between groups at days 7 and 14 of culture, and lowest values for the *GFP* controls at day 21 (Fig. 6b).

For quantitative comparison and assessment of extracellular matrix synthesis among the transduced groups, GAG levels in the pellet cultures during 21 days in culture were determined (Fig. 6c). The chondrogenic aggregate groups *SOX9*, *SOX9* + *TGFB1*, *TGFB1* alone, and *SOX9* + *IGF1* showed elevated levels of GAG compared to the non-chondrogenic *GFP* control and the *IGF1* alone group during the time-course of 21 days. Differences that reached statistical significance (*p <* 0.05) are marked with asterisks, with the *TGFB1* only modified aggregated being the group that reached the significance levels earliest day 3 of culture (Fig. 6c). At days 14 and 21, no significant differences between levels of GAG among the chondrogenic groups SOX9, *SOX9* + *TGFB1*, *TGFB1* alone, and *SOX9* + *IGF1* could be resolved (Fig. 6c).

We analysed ALP activity within the different groups of aggregates as a measure of hypertrophy. ALP was found markedly elevated at all time points in the *SOX9* + *TGFB1* pellets and even higher in pellets receiving *TGFB1* alone, compared to the *GFP* controls. There were no significant differences between *GFP* controls, *SOX9*, *SOX9* + *IGF1*, and *IGF1* alone during the evaluated time-course of 21 days (Fig. 6d).

### Real-time RT-PCR analyses - Chondrogenic and hypertrophic marker gene expression

**Fig. 7:**
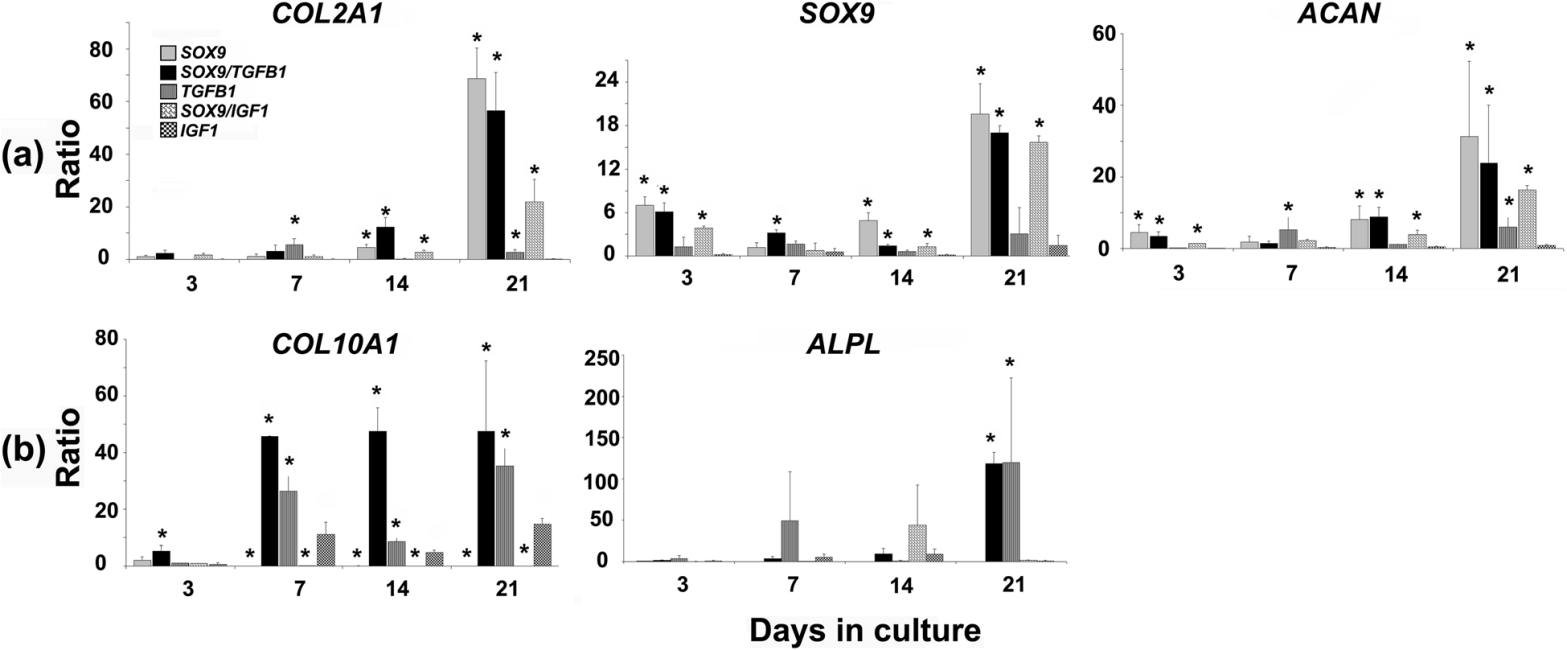
Temporal gene expression profiles determined by real time RT-PCR in MSC pellet cultures genetically modified with *SOX9* compared to *TGFB1* or *IGF1* alone or in combination. Profiles of temporal gene expression determined by real time RT-PCR in MSC pellet cultures after chondrogenic induction using adenoviral vectors encoding *SOX9*, *SOX9* + *TGFB1*, *TGFB1*, *SOX9* + *IGF1* or *IGF1*. Genes analysed include collagen (COL) type II (*COL2A1*), *SOX9*, aggrecan (*ACAN*), COL type X (*COL10A1*), alkaline phosphatase (*ALPL*). Primer sequences and product size are listed in Table 1, with elongation factor 1α (*EEF1A1*) serving as housekeeping gene and internal control. For each marrow preparation/patient, treatment group and time point RNA was extracted from 10 pellets. Values of real-time RT-PCR analyses represent mean expression ratios ± SD normalised to the expression levels of the housekeeping gene *EEF1A1* and the *GFP* controls as determined by the ΔΔCT method, with significant differences *(p <* 0.05) relative to values from the non-chondrogenic *IGF1* group being marked with asterisks (*).

For a more accurate quantification of the expression of marker genes associated with chondrogenesis and hypertrophy, real-time RT-PCR analyses were performed for selected genes (*COL2A1*, *SOX9*, *ACAN*, *COL10A1* and *ALPL*). Expression of marker genes associated with chondrogenesis (*COL2A1*, *SOX9*, *ACAN*) was upregulated in all chondrogenic groups with the highest levels being visible for the *SOX9*, the *SOX9* + *TGFB1* and the *SOX9* + *IGF1* groups especially at day 21 of culture (Fig. 7a). Notably, high levels of *SOX9* in these groups at day 3 may reflect transgene expression and not only chondrogenic induction.

Genes associated with chondrogenic hypertrophy such as *COL10A1* and *ALPL* were more significantly upregulated in the *TGFB1* and *SOX9* + *TGFB1* groups compared to the *SOX9* and the *SOX9* + *IGF1* groups over time, relative to the marker gene controls (Fig. 7b). These results suggest that *TGFB1* rather than *SOX9* induced a significant hypertrophic response in MSC aggregates at the mRNA level.

## Discussion

Although the potential advantages of MSCs as agents of cartilage regeneration have been recognised for a long time, their deployment in this regard remains problematic [34–37]. Among the challenges is the need to provide a sustained chondrogenic signal to the MSCs while preventing terminal, hypertrophic differentiation with consequent apoptosis and endochondral ossification.

We and others have explored the use of gene transfer as a means of providing self-sustaining chondrogenic stimuli to MSCs [4, 6–8, 23, 38–40]. A number of transgenes, such as those encoding TGF-β, BMP-2 and BMP-4 have shown success in this endeavour, but in each case there was progression towards hypertrophy [6, 7].

The present study shows that transfer of cDNA encoding *SOX9* is more effective than *TGFB1* in promoting chondrogenesis in pellet culture. Moreover, unlike *TGFB1*, *SOX9* does this without inducing the markers of hypertrophic differentiation during the 21-day incubation period. Notably, ALP staining in the *TGFB1* group was highest at day 10 of culture, whereas the expression at day 21 was low, which might reflect a phaseal upregulation during osteogenic pathways which are seen during osteogenesis of mesenchymal cells [1, 9]. Intriguingly, co-transduction of MSCs with *SOX9* and *TGFB1* reduced the effectiveness of *SOX9* both in terms of promoting chondrogenesis and preventing the emergence of markers of hypertrophy. IGF-1 alone, as noted previously, induced neither chondrogenesis nor hypertrophy [6]. Like in previous work [6] the combination groups (*SOX9* + *TGFB1* or *SOX9* + *IGF1*) received twice the amount of infectious viral particles, which resulted in comparable amounts of transgene expression for each transgene, however, synergistic effects on the chondrogenic phenotype have not been observed.

Previous authors have studied *SOX9* as an agent of chondrogenesis, using MSCs, de-differentiated chondrocytes and iPS cells in this regard [41–43]. Although the results of these studies are largely in line with our data, there are some discrepancies. Kupcsik et al., for instance, only noted increased GAG synthesis in response to *SOX9* when the cells were also mechanically stimulated [44]. This may reflect the MSC culture conditions as their study employed human MSCs seeded into a hydrogel whereas we used pellet cultures. Other authors have reported a chondrogenic response only when *SOX9* was co-transferred with *SOX5* and *SOX6* (*SOX* trio) [45]. Nevertheless, *SOX9* clearly merits further study in the context of chondrogenesis and cartilage repair. In vivo experiments support this assessment. Preliminary studies using rabbits suggest a role in cartilage repair for MSCs transduced with Ad.*SOX9* and seeded onto a polyglycolic acid scaffold [15].

Because *SOX9* is an intracellular protein it is difficult to deliver to target cells by traditional methods. Gene transfer overcomes this barrier. Future clinical translation of this technology will require an appropriate vector. The recombinant adenovirus used in the present study is straightforward to produce and provide transgene expression for 2–3 weeks, which may be sufficient to provoke a sustainable regenerative response. Moreover, our data suggest that the effects of *SOX9* are sustained and amplified by autocrine effects, as revealed by the persistence of *SOX9* expression in cells after expression from the *SOX9-GFP* fusion transgene has ceased.

Adenovirus has been widely used in human gene therapy trials which showed this vector to stimulate innate and humoral immune responses that are disadvantages for systemic delivery [46–48]. However, local delivery of the type that would be used for cartilage repair is unlikely to be problematic. Adeno-associated virus (AAV) is increasingly becoming the vector of choice for human gene therapy [43, 49]. Cucchiarini and Madry have successfully used AAV to deliver *SOX9* to chondrocytes and MSCs, with results consistent to those reported in the present paper [50]. A clinical trial in which AAV is injected into joints with osteoarthritis was recently initiated (ClinicalTrials.gov Identifier: NCT02790723).

Daniels et al. could show that *SOX9* overexpression via AAV gene transfer to human osteoarthritic articular chondrocytes leads to a significant production of ECM components like proteoglycans and COL type II without affecting the cell proliferation [26]. These findings are consistent with our data showing SOX9 as an effective inducer of chondrogenesis. Interestingly, combined AAV gene transfer of *TGFB* and *SOX9* in bone marrow aspirates could induce chondrogenesis and reduce hypertrophic differentiation [27]. A finding that could not be confirmed by our data showing that genes associated with chondrogenic hypertrophy such as *COL10A1* and *ALPL* were more significantly upregulated in the *TGFB1* and *SOX9* + *TGFB1* groups compared to the *SOX9* group.

## Conclusion

Adenoviral *SOX9* gene transfer induces chondrogenic differentiation of human primary MSCs in pellet culture more effectively than *TGFB1* gene transfer with lower levels of chondrocyte hypertrophy after 3 weeks of in vitro culture. This technology might be harnessed to develop methods for allowing sustained chondrogenesis while preventing hypertrophic differentiation, thus leading to improved cartilage repair.

## Data Availability

The datasets used and/or analysed during the current study are available from the corresponding author on reasonable request.

## Abbreviations

*ACAN*: Aggrecan core protein gene
Ad: Adenoviral vector
ALP: Alkaline phosphatase
*ALPL*: ALP gene
ATP: Adenosine 5 triphosphate
BMP: Bone morphogenetic protein
COL: Collagen
*COL10A1*: COL10 gene
*COL2A1*: COL2 gene
CS: Chondroitin sulphate
DMMB: Dimethylmethylene blue
*EEF1A*: Elongation factor 1 alpha gene
GAG: Glycosaminoglycan
*GFP*: GFP gene
GFP: Green fluorescent protein
*IGF1*: IGF-1 gene
IGF-1: Insulin like growth factor 1
*IHH*: IHH gene
IHH: Indian hedgehog
iPS: Induced pluripotent stem cell
LUC: Firefly luciferase
*LUC*: LUC gene
MSC: Mesenchymal stem cell
RUNX2: Runt-related transcription factor 2
*SOX9*: SOX9 gene
SOX9: SRY (sex determining region Y) - box 9
*TGFB1*: TGF-β1 gene
TGF-β1: Transforming growth factor β1
